# A Cascaded Convolutional Neural Network for Assessing Signal Quality of Dynamic ECG

**DOI:** 10.1155/2019/7095137

**Published:** 2019-10-20

**Authors:** Qifei Zhang, Lingjian Fu, Linyue Gu

**Affiliations:** Helowin Medical Technology, Hangzhou 310012, China

## Abstract

Motion artifacts and myoelectrical noise are common issues complicating the collection and processing of dynamic electrocardiogram (ECG) signals. Recent signal quality studies have utilized a binary classification metric in which ECG samples are determined to either be clean or noisy. However, the clinical use of dynamic ECGs requires specific noise level classification for varying applications. Conventional signal processing methods, including waveform discrimination, are limited in their ability to remove motion artifacts and myoelectrical noise from dynamic ECGs. As such, a novel cascaded convolutional neural network (CNN) is proposed and demonstrated for application to the five-classification problem (low interference, mild motion artifacts, mild myoelectrical noise, severe motion artifacts, and severe myoelectrical noise). Specifically, this study finally categorizes dynamic ECG signals into three levels (low, mild, and severe) using the proposed CNN to meet clinical requirements. The network includes two components, the first of which was used to distinguish signal interference types, while the second was used to distinguish signal interference levels. This model does not require feature engineering, includes powerful nonlinear mapping capabilities, and is robust to varying noise types. Experimental data are composed of private dataset and public dataset, which were acquired from 90,000 four-second dynamic ECG signals and MIT-BIH Arrhythmia database, respectively. Experimental results produced an overall recognition rate of 92.7% on private dataset and 91.8% on public dataset. These results suggest the proposed technique to be a valuable new tool for dynamic ECG auxiliary diagnosis.

## 1. Introduction

**Figure 1:**
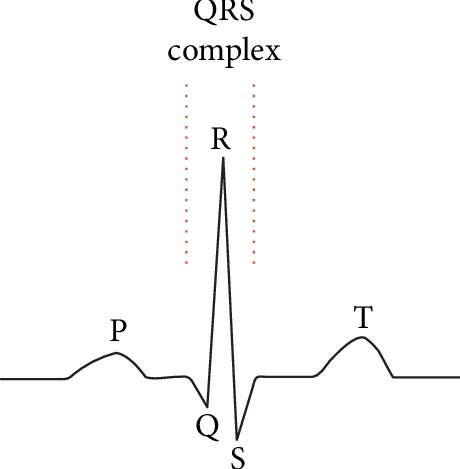
A sample ECG signal.

**Figure 2:**
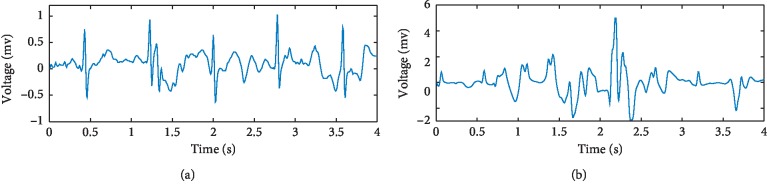
(a) Mild motion artifacts and (b) severe motion artifacts.

**Figure 3:**
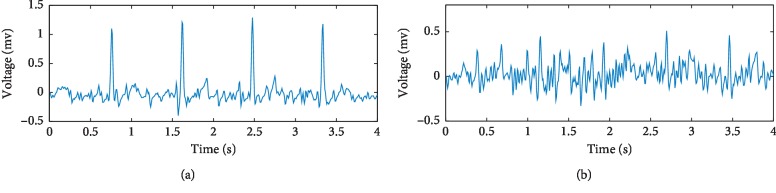
(a) Mild myoelectrical noise and (b) severe myoelectrical noise.

The electrocardiogram (ECG) signal shown in Figure 1 is composed of a P wave, a QRS wave, and a T wave. Its amplitude is in the range of 0.05–5 mv, and its frequency ranges from 0.1–60 Hz. Dynamic ECGs, collected using a Holter monitor, provide a record of ECG signals over extended periods of time [1]. These are commonly used in the diagnosis of arrhythmia and myocardial ischemia, as well as 24-hour monitoring of daily patient activities via an ECG. In contrast to dynamic ECGs, routine ECGs are collected from patients in a resting state. As such, baseline drift, motion artifacts, and myoelectrical noise are common in dynamic ECGs. Baseline drift can be removed using a high-pass filter. However, motion artifacts are more problematic and are caused by changes in contact resistance resulting from movement of the body. As shown in Figure 2, signals containing mild and severe motion artifacts manifest as irregular abrupt waves with frequencies below 7 Hz. Myoelectrical noise, caused by muscle activity, is also difficult to remove. As shown in Figure 3, signals containing mild and severe myoelectrical noise are primarily characterized by rapidly changing blur waves. These vary in amplitude with frequencies in the range of 30–300 Hz. Besides, motion artifacts and myoelectrical noise can also overlap on the ECG spectrum, leading to the potential for false diagnosis.

Clinical dynamic ECGs require high accuracy for these types of interference measurements. As such, the primary objective of this study is to quantify the degree of interference present in a given signal. This would optimize the use of signals with preferable interference levels, for improved diagnosis of specific diseases. We intend to divide dynamic ECG signals into three levels: low, mild, and severe. Low-interference signals could be used for the diagnosis of arrhythmia or myocardial ischemia, mild-interference signals could be confidently used for heartbeat or heart rate variability (HRV) measurements, and signals containing severe levels of interference could be safely ignored to prevent a false diagnosis.

The 2011 PhysioNet/Computing in Cardiology (PCinC) Challenge [2] proposed the development of an efficient algorithm, capable of running on a mobile phone, for estimating whether routine ECG signals were acceptable or not. To date, various studies have focused on developing a signal quality assessment index (SQI) that extracts actual features. Behar et al. [3] extracted 7 SQIs from the PCinC database, MIT-BIH arrhythmia database [4], and MIMIC II database [5] and used a support vector machine (SVM) to assess ECG quality for both normal and abnormal rhythms. The study was focused on preventing the misidentification of motion artifacts or myoelectrical noise as arrhythmia in ICU ECG monitoring. Zhang et al. [6] used multiscale entropy as an SQI, since signals with varying signal-to-noise ratios (SNRs) exhibit differing multiscale entropy levels. Orphanidou et al. [7] extracted 4 SQIs from the PCinC database to determine whether ECG signals were acceptable for clinical use, achieving a sensitivity of 94% and a specificity of 97%. Johnson et al. [8] proposed a model for heartbeat detection which extracted 2 SQIs from the PCinC database, achieving a sensitivity of 94.07% and a specificity of 89.03%. Besides, Redmond et al. [9] classified the ECG quality into three categories based on the determinability of heart rate (HR): good (HR is easy to determine); average (HR is difficult, but possible to determine); bad (HR cannot reliably be determined), which reached an accuracy of 78.7% on their own test data. Li et al. [10] divided ECG signal quality into five levels according to signal-to-noise ratio (SNR). A total of 13 SQIs derived from ECG waveform segments were input to a support vector machine (SVM) and used to classify a simulated dataset. Tests conducted on the MIT-BIH arrhythmia database produced an accuracy of 88.07% as well. While these studies could also be applied to the classification of dynamic ECGs, an effective assessment of signal quality is required in clinical applications because dynamic signals often include motion artifacts and myoelectrical noise.

ECG signal classification has also been attempted using classical machine learning methods. However, such techniques require time-consuming feature engineering to identify the available features. For example, Zhang et al. [11] input certain nonlinear features, extracted from ECG signals, into machine learning algorithms such as SVM and random forest (RF). This was done to provide a comparison and determine ECG signal suitability. Such classical machine learning approaches include several inherent limitations, such as fewer training samples, poor generalization capabilities, and tedious feature extraction. In contrast, deep learning techniques [12] do not require complex feature engineering and have successfully been applied to a variety of fields including image recognition [13], speech recognition [14], and machine translation [15]. However, due to the scarcity of ECG data, there have been few studies on the application of deep learning to the classification of dynamic ECG interference.

This study proposes a dynamic ECG quality assessment model that extracts ECG signal features and evaluates signal quality using a cascaded convolutional neural network (CNN). This model was divided into two stages. The first stage, which included a subnetwork, was used to distinguish signal interference type. The second stage consisted of two subnetworks that were similar to subnetwork in the first stage. It was used to further estimate the degree of signal interference. This model was trained and tested until dynamic ECG signals in the dataset had been classified into one of five classes (low interference, mild myoelectrical noise, severe myoelectrical noise, mild motion artifacts, and severe motion artifacts). Furthermore, we categorized the classification results into three levels: low (low interference), mild (mild myoelectrical noise and mild motion artifacts), and severe (severe myoelectrical noise and severe motion artifacts).

The contributions of this study can be summarized as follows:

1. We propose a novel cascaded CNN that includes two components, the first of which was used to distinguish signal interference type, while the second was used to assess signal interference levels. In other words, the model conducts a five-classification task on the acquired dataset.
2. Dynamic ECG signals were divided into three levels (low, mild, and severe) to meet clinical requirements. Low-interference signals could be used for the diagnosis of arrhythmia or myocardial ischemia. Mild-interference signals could be confidently used for heartbeat or heart rate variability (HRV) measurements. Signals containing severe levels of interference could be safely ignored to prevent a false diagnosis.
3. Low-interference signals were defined, according to clinical requirements, as having little interference levels. Mild and severe interference were defined by the clear visibility of an R wave. As such, when preparing dataset, we quantified the standards for data labelling.
4. Experimental results showed that our model was superior to others. As a result, the model achieved promising performance for dynamic ECG auxiliary diagnosis.

The remainder of this paper is organized as follows.  Section 2 introduces the data and methodology in additional detail.  Section 3 details the experimental configurations, results, and discussions.  Section 4 concludes the paper and describes possible directions for future research.

## 2. Materials and Methods

### 2.1. Private Dataset

Considering a large number of data were required to train and validate our model, we prepared private dataset by ourselves. The process of preparing dataset involved data collection, data preprocessing, and data labelling. Specially, preprocessing raw dynamic ECGs was helpful to differentiate these five categories. The data were recorded, labelled, and reviewed by two cardiologists. Technically, we also presented the rules of data labelling for reference.

#### 2.1.1. Data Collection

The ECG recordings with standard 12-lead channels were collected using TE-9000Y, a Holter monitor made in Helowin medical technology company in China. The ECGs were digitized at 128 samples per second per channel over a 10 mV range. In case of individual diversity, 2,100 subjects, from the Hospital of Tonglu County, Zhejiang Province in China between 2014 and 2017, included 1,500 outpatients (about 60% men aged 23 to 85 years and 40% women aged 25 to 89 years) and 600 inpatients (about 70% men aged 32 to 86 years and 30% women aged 28 to 87 years). Eventually, we acquired 2,100 24-hour II-lead dynamic ECG signal recordings from those subjects.

#### 2.1.2. Data Pre-Processing

A second-order Butterworth filter was used in the preprocessing steps. High-frequency noise above 40 Hz and baseline drift below 0.5 Hz were filtered out.

#### 2.1.3. Data Labelling

The dynamic ECG signals of five categories were intercepted from recordings and labelled, respectively. For the sake of the robustness of the model, some tricks would be needed inevitably. For low interference, we preferred to select those signals with cardiac arrhythmias, myocardial ischemia, etc. Exhibiting varied heart rates, etc., signals with corresponding moderate interferences were first selected for mild myoelectric interference and mild motion artifact as well. Because severe interferences covered the signals thoroughly, we intercepted data at random for severe myoelectric interference and severe motion artifact.

We selected 10 4-second non-overlapping excerpts for the category of the low interference from each of 1,500 recordings (from 1,500 outpatients). And, then 25 4-second non-overlapping segments were picked for category of low interference from each of 600 recordings (from 600 inpatients) to include less common but clinically significant arrhythmias, because low interference exists abundantly in each recording. Moreover, compared with recordings of outpatients, those of inpatients have less interference. For the mild myoelectric interference, 30 4-second non-overlapping excerpts were obtained from each of 500 recordings, which had at least 10-minute mild myoelectric interference and selected from 1,500 recordings (from 1,500 outpatients). For the mild motion artifact, 25 four-second non-overlapping excerpts were obtained from each of 600 recordings, which presented at least 10-minute mild motion artifacts and was selected from 1,500 recordings. For the severe myoelectric interference, 75 four-second non-overlapping excerpts were obtained from each of 200 recordings, which had at least 10-minute severe myoelectric interference and selected from 1,500 recordings. For the severe motion artifact, 50 four-second non-overlapping excerpts were obtained from each of 300 recordings, which had at least 10-minute severe motion artifacts and was selected from 1,500 recordings as well.

In conclusion, the dataset included 90,000 II-lead dynamic ECG signals, with a 4-second duration and a sampling rate of 128 Hz. This set consisted of 30,000 low interference (including about 9,000 signals with cardiac arrhythmias, myocardial ischemia, etc.), 15,000 mild motion artifacts (including about 800 signals with varied heart rates, etc.), 15,000 severe motion artifacts, 15,000 mild myoelectric interference (including about 1,000 signals with varied heart rates, etc.), and 15,000 severe myoelectric interference samples.

#### 2.1.4. Rules of Data Labelling

We selected a segment with 4-second duration, based on two facts: after preprocessing, we found in a large number of clinical data that the possibility of two or more interference patterns (excepting low interference) coexisting within a 4-second period is extremely small and at least one cardiac rhythm cycle is included. As such, only one type of interference that dominated the 4-second duration was assigned as label of each dynamic ECG sample. That is to say, if an interference duration presented in the signal exceeded 2 seconds, it would be the label. The rules of labelling data were defined in detail as follows:

1. If the duration exceeded 2 seconds and the amplitude of the noise was so sufficiently small that the P, QRS, and T waves are clear, it was considered low interference
2. If the signal had myoelectrical noise and the maximum amplitude of the noise was less than half the height of the clear R-wave in a heartbeat cycle (with a duration exceeding 2 seconds), it was mild myoelectrical noise
3. If the signal had motion artifacts and the maximum amplitude of the noise was less than half the height of the clear R-wave in a heartbeat cycle (with a duration exceeding 2 seconds), it was classified as mild motion artifacts
4. If the signal had myoelectrical noise and the maximum amplitude of the noise exceeded half the height of the clear R-wave in a heartbeat cycle (with a duration exceeding 2 seconds), it was severe myoelectrical noise
5. If the signal had motion artifacts and the maximum amplitude of the noise exceeded half the height of the clear R-wave in a heartbeat cycle (with a duration exceeding 2 seconds), it was classified as severe motion artifacts

### 2.2. Public Dataset

Being used as the validation dataset as well, the MIT-BIH arrhythmia database [4] includes 48 half-hour excerpts of two-channel ambulatory ECG recordings with arrhythmia reference annotations. Usually, the first channel is the modified limb lead II and the second is a precordial lead V1. The recordings have a diagnostic band width of 0.1–100 Hz and were digitized at 360 Hz. Therefore, the channel of lead II was selected and was downsampled to 128 Hz to match our study. A 4-second non-overlapping window was used to segment the ECG data into different classes. The dataset was reannotated to include the five classes according to the rules of data labelling in Section 2.1. A summary of the number of ECG segments of each class is found in Table 1.

### 2.3. Time-Frequency Spectrum of Dynamic ECG Signals

Short-time Fourier transforms (STFTs) [16] are a classical time-frequency analysis method which describes frequency domain characteristics by analysing a segment of a signal in a specific time window. The frequency characteristics of different window function types vary in the short-time Fourier transform. As such, both the time and frequency resolution must be considered.

**Figure 4:**
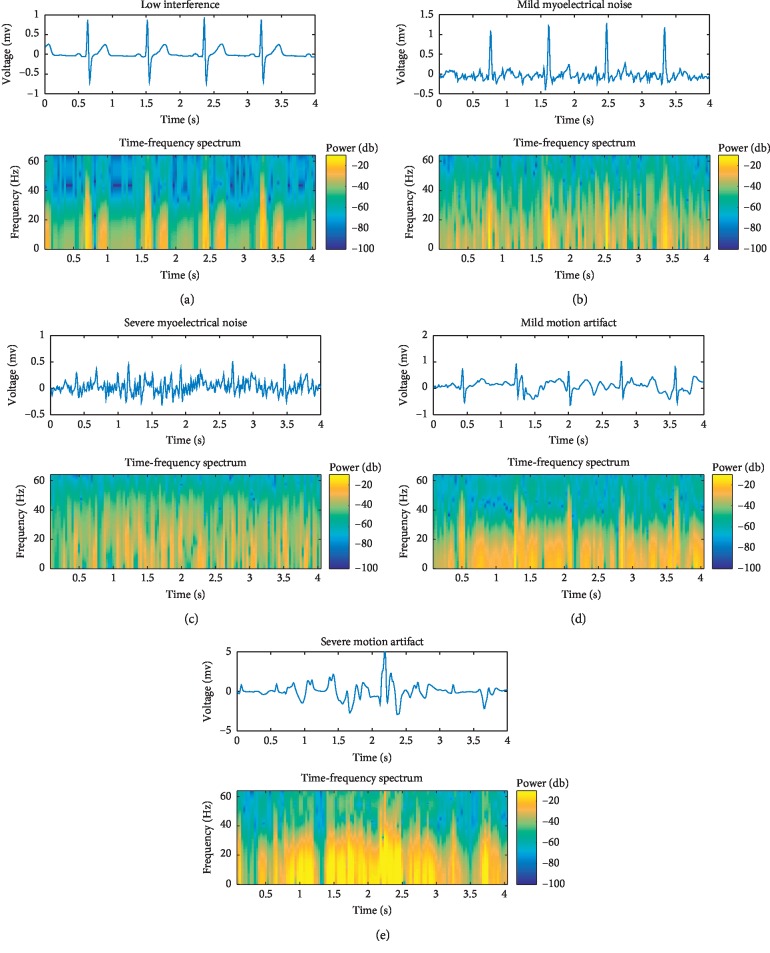
The time-frequency spectrum for five types of dynamic ECG signals.

We applied the time-frequency spectrum of our dynamic ECG signal to a convolutional neural network model in a process similar to speech recognition [17–20]. This study utilized an 8-point symmetric Hamming window because it reduced spectrum leakage and acquired better time-frequency details after multiple trials. As a result, after the short-time Fourier transform, dynamic ECG signals (with a size of 1 × 512) were converted into the time-frequency spectrum (257 × 63). The time domain signal and time-frequency spectrum for five types of dynamic ECGs are shown in Figure 4. Low interference signals were identified as having negligible interference. Myoelectrical noise and motion artifacts had different levels of energy distribution and wave shapes. The signatures in time and frequency domain that were characteristic of low interference, motion artifacts, and myoelectrical noise were distinguishable. Additionally, signals with low interference exhibited clear wave properties, signals with mild electrical noise or mild motion artifacts exhibited R waves, and signals with severe electrical noise or motion artifacts included significant interference that overlapped on the ECG spectrum.

### 2.4. Convolutional Neural Networks

CNNs [21] are feedforward neural networks, containing deep neural network models with a convolutional layer and a pooling layer. This special structure facilitates unique characteristics such as local perception, weight sharing, and pooling. Local perception involves a convolution kernel operating in a local rectangular region within an image, in order to acquire a feature map. Weight sharing involves the sharing of weights and biases in a convolution kernel for each feature map. Pooling is a descending sampling operation in the feature map, with a goal of reducing and summarizing the acquired feature map. Two conventional choices, average pooling and maximum pooling, acquire an average or maximum of small rectangular blocks within the feature map. The size of these data can be reduced without affecting the extracted features. During supervised learning, the output from these layers was flattened into a one-dimensional vector (after several convolutional and max-pooling layers) for input into the fully connected neural network. This usually included one or more fully connected layers for classification.

**Figure 5:**
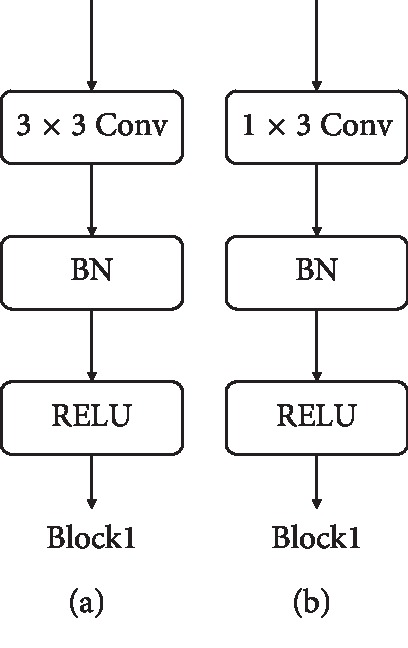
Two types of convolutional neural networks (block1 and block2).

Several studies have demonstrated that small convolution kernels achieve improved recognition accuracy in CNNs [22–24]. Lin et al. [25] demonstrated that a convolution kernel with a size of 1 × 1, acting as a cross-channel aggregation, could further reduce dimensionality and the number of parameters. However, this process had little effect on recognition accuracy. This 1 × 1 convolution kernel was also applied to reduce dimensionality in our model. Hinton et al. [26] demonstrated that “dropout” reduced overfitting by randomly omitting part of the neuron connections in each training case, which proved to be an efficient way of performing model averaging with neural networks. This “dropout” was also applied to our model. Ioffe and Szegedy [27] showed that the inclusion of batch normalization (BN) led to improved recognition results. Glorot et al. [28] demonstrated that using a RELU as a nonlinear activation function in deep convolutional neural networks could eliminate problems caused by vanishing gradients, which is beneficial for model convergence. As such, in the dynamic ECG quality assessment model, the network was stacked with a block labelled “block1” (including a 3 × 3 convolutional layer) and block labelled “block2” (including a 1 × 3 convolutional layer). The BN layer and RELU activation function layer are also shown in Figure 5.

### 2.5. A Dynamic ECG Signal Quality Assessment Model

This study proposes a novel dynamic ECG quality assessment model to compensate for limitations such as a lack of fine-grained recognition for clinical requirements, shortages of signal processing methods, and complex feature engineering. Through the model, ECG signals will be classified into five classifications, which are then divided into three levels.

**Figure 6:**
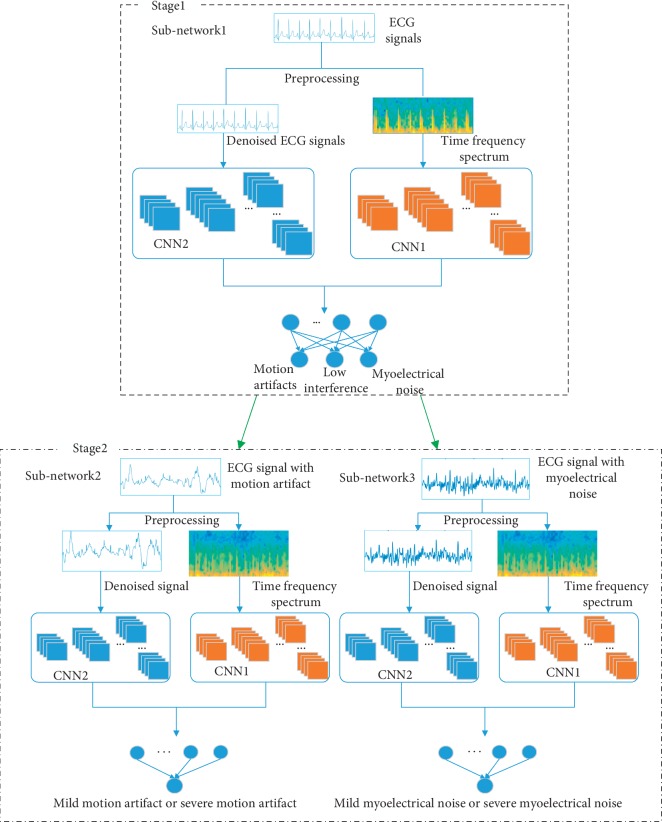
A dynamic ECG signal quality assessment model.

The cascaded CNN model includes two stages, as shown in Figure 6. The first stage, which included subnetwork1, was used to distinguish signal interference type. After the preprocessing of a single lead ECG signal, a time-frequency spectrum was acquired using a short-time Fourier transform. The time-frequency spectrum was then input to the convolutional neural network (CNN1) for feature extraction. Meanwhile, the preprocessed ECG signal was input to another convolutional neural network (CNN2) for feature extraction. The features extracted from CNN1 and CNN2 were combined and input to a fully-connected neural network that included a fully-connected layer and a softmax layer for classification into three classes (low interference, myoelectrical noise, and motion artifacts). The second stage consisted of two subnetworks that were similar to subnetwork1 in the first stage. It was used to further estimate the degree of signal interference. Signals containing motion artifacts, as identified by the first stage, were input to subnetwork2 in the second stage to estimate whether the motion artifacts were mild or severe. Signals containing myoelectrical noise, as identified by the first stage, were input to subnetwork3 in the second stage to determine whether the noise was mild or severe. Finally, ECG signals input into our model would be classified into five classes.

In the first stage of Figure 6, for the subnetwork1, the frequency spectra produced by a short-time Fourier transform of the preprocessed dynamic ECG signal set ({[ecg_*i*_, *y*_*i*_]}, *i*=1,2,…, *n*) were input to the convolutional neural network CNN1 model, resulting in the feature vector *M*_*i*_ (*M*_*i*_ ∈ *R*^*m∗*1^). The dynamic ECG signals were directly input to another convolutional neural network CNN2 model as one-dimensional time series to acquire the feature vector *N*_*i*_ (*N*_*i*_ ∈ *R*^*n∗*1^). The combined vectors *M*_*i*_ and *N*_*i*_ were then input into a neural network including a fully connected layer and softmax layer. Finally, the output vector *O*_*i*_ was acquired. This process can be described as follows:

(1) $$\begin{array}{c} \left\{\begin{array}{c} M_{i}=f_{\text{CNN}1}\left(\text{stft}\left({\text{ecg}}_{i}\right),\mathrm{W1},\mathrm{b1}\right),\\ N_{i}=f_{\text{CNN}2}\left({\text{ecg}}_{i},\mathrm{W2},\mathrm{b2}\right), \end{array}\right.\\ O_{i}=g\left(\left[M_{i};N_{i}\right],\mathrm{W3},\mathrm{b3}\right), \end{array}$$

where stft(ecg_*i*_) denotes the value of the dynamic ECG signal after a short-time Fourier transform, **W1**, **W2**, and **W3** denote weight matrices in the neural network, **b1**, **b2**, and **b3** denote biases in the neural network, *f*_CNN1_ is the feature extraction process for the CNN1 convolutional neural network, *f*_CNN2_ is the feature extraction process for the CNN2 convolutional neural network, and *g* denotes feature mapping of the fully connected neural network which contains a fully connected layer and softmax layer.

In the first stage, the gradient descent method of error backpropagation was used during network training. There are 3 neurons in the last layer of the softmax layer, since the first network stage classifies data into three classes. A cross entropy loss function could then be constructed using the true values of the samples *y*_*i*_ and the predicted values of the network *O*_*i*_ as follows:

(2) $$\begin{array}{c} \text{loss}=-\overset{3}{\underset{j=1}{\sum }}\left[y_{i}^{j}*\log\;O_{i}^{j}+\left(1-y_{i}^{j}\right)\log\left(1-O_{i}^{j}\right)\right],\;j=1,2,3, \end{array}$$

where *O*_*i*_^*j*^ denotes the predicted result of the *j*^th^ class and the *i*^th^ sample and *y*_*i*_^*j*^ denotes the true result of the *j*^th^ class and *i*^th^ sample.

The second stage consisted of two subnetworks. Subnetwork2 was used for the binary classification of mild motion artifacts and severe motion artifacts. Subnetwork3 was used for the binary classification of mild myoelectrical noise and severe myoelectrical noise. Subnetwork2 and subnetwork3 had the same convolutional structure as the subnetwork1 in the first stage, described previously except softmax layer.

## 3. Experiments

In the process of experiments, two models, including baseline model and our cascaded CNN model, were defined. For the purpose of demonstrating that our model was able to improve the performance of assessing quality of ECGs, we carried out two groups of experiments. The one was that two models were trained and tested on private dataset for comparisons. The other was that after being trained on private dataset, those two models were tested on public dataset for comparing with the previous studies [3, 10].

### 3.1. Experimental Platform and Evaluation Index

The experimental platform consisted of three IBM servers whose configuration included an Intel Xeon E5-2650 2.8 GHz CPU, 16 GB of DDR3 memory, an 8 GB GTX1080 GPU, and a 64 bit Centos operating system. MATLAB 2017a was used for data processing, and the neural network code was written in the TensorFlow v1.10 framework based on the Python language. The following indices were used to measure the performance of the recognition model.

Sensitivity (Se) was defined as the percentage of true positive samples among samples which were judged to be true positive and false negative by the model. It was calculated using Se = TP/(TP + FN). Specificity (Sp) was defined as the proportion of good quality signals that have been correctly identified as acceptable. It was calculated as Sp = TN/(FP + TN). Accuracy (Ac) measured the percentage of all true samples among all samples recognized by the model. It was Ac = (TP + TN)/(TP + TN + FP + FN). These statistical measures were calculated by the number of true positives (TP), true negatives (TN), false positives (FP), and false negatives (FN).

### 3.2. Baseline Model

#### 3.2.1. Baseline Model

The experiment conducted in our study established a baseline model for comparative assessment. We used a single convolutional neural network to directly classify five dynamic ECG signal types, using a universal methodology applied to similar problems [29–33]. The configuration of this baseline model is shown in Table 2.

The input in Table 2 consisted of preprocessed dynamic ECG signals with a size of 1 × 512. The convolution process was stacked by block2 shown in Figure 5. The format was implemented as “{number of blocks}_block2_{number of feature maps}.” The pool layer utilized maximum pooling, the format was structured as “Max_pooling_{size},” and the final convolutional layer (Conv_1 × 1_32) was a kernel of size 1 × 1 with 32 feature maps. This fully connected layer (Fc_128) included 128 neurons, while the softmax layer contained 5 neurons.

#### 3.2.2. Training Details

This experiment included 10,000 test data samples, 2,000 validation data samples, and a set of training data on private dataset. The following parameters were used in the experiments. The Adam optimizer [34] was included for training the model. The initial learning rate was set to 0.03, the decay rate was 0.50, and the decay step was 10,000. Weights and biases were initialized using a method proposed in a previous study [35]. The training phrase included 100 epochs with minibatches of 100 sample signals.

### 3.3. Cascaded CNN Model

#### 3.3.1. Cascaded CNN Model

The cascaded CNN model shown in Figure 6 is primarily composed of two networks: CNN1 and CNN2. Each of these networks was included in the experiment. The specific parameters are shown in Table 3.

Table 3 details a time-frequency spectrum of size 257 × 63, acquired from a preprocessed dynamic ECG signal using a short-time Fourier transform input to the CNN1 network. The convolutional layers were stacked with block1 (Figure 5) using the format “{number of blocks}_block1_{number of features}.” The pool layer used maximum pooling, and the format was structured as “Max_pooling_{size}.” The final convolutional layer (Conv_1 × 1_32) included a kernel of size 1 × 1 with 32 feature maps. The CNN2 network is similar to CNN1, and its input data consisted of preprocessed dynamic ECG signals with a size of 1 × 512. The fully connected network included Fc_128, which consisted of 128 neurons and a softmax layer. The “dropout” was applied to Fc_128.

#### 3.3.2. Training Details

The cascaded network shown in Figure 6 contains 3 subnetworks including subnetwork1, subnetwork2, and subnetwork3. These 3 subnetworks were first trained separately. The cascaded network made up of 3 subnetworks was then tested. The private dataset consisted of 30,000 low interference, 15,000 mild motion artifacts, 15,000 severe motion artifacts, 15,000 mild myoelectric interference, and 15,000 severe myoelectric interference samples. A set of 10,000 samples were selected for model testing. The remaining set included 28,000 low interference, 13,000 mild motion artifacts, 13,000 severe motion artifacts, 13,000 mild myoelectric interference, and 13,000 severe myoelectric interference samples. The data in subnetwork1 consisted of low interference (28,000 samples), motion artifacts (13,000 mild and 13,000 severe), and myoelectric interference (13,000 mild and 13,000 severe) samples. This set was split into 78,000 training and 2,000 validation samples. Subnetwork2 included 13,000 mild motion artifact and 13,000 severe motion artifact samples, which were split into 25,000 training and 1,000 validation samples. Subnetwork3 included 13,000 mild myoelectric interference and 13,000 severe myoelectric interference samples, which were split into 25,000 training and 1,000 validation samples. The 3 subnetwork parameters were used in the experiments. The Adam optimizer [34] was used for training. The initial learning rate was set to 0.02, the decay rate was 0.50, and the decay step was 10,000. The probability of “dropout” was 50%. Weights and biases were initialized using a method proposed in a previous study [35]. The training phase included 80 epochs with minibatches of 100 sample signals.

### 3.4. Experimental Results

#### 3.4.1. Results on Private Dataset

Baseline and our cascaded CNN model were trained and tested on private dataset. As shown in Table 4, optimal performance for the baseline model reached 88.3%, using the parameters listed in Table 2 (W1 = 1, W2 = 2, and W3 = 5). Setting the parameters as N1 = 1, N2 = 2, and N3 = 5 (Table 3) produced an overall Ac of 92.7%, which was better than that in baseline model. The Se and Sp reached 95.4% and 98.4% (low interference), 91.1% and 98.2% (mild myoelectrical noise), 92.9% and 98.4% (severe myoelectrical noise), 92.3% and 97.7% (mild motion artifacts), and 91.8% and 98.3% (severe motion artifacts), respectively. This suggests the proposed model outperformed the baseline model in each class.

#### 3.4.2. Results on Public Dataset

After training those two models on private dataset, we evaluated them on public dataset further. As shown in Table 5, results of four models, including 7-SQI SVM [3], 13-SQI SVM [10], baseline model, and our cascaded model, are listed for comparisons. An Ac of 94.6%, a Se of 86.3%, and a Sp of 94.8% were gained using 7-SQI SVM to conduct binary task. Seen from the results of five classifications (clean, minor, moderate, severe, and extreme noise) using 13-SQI SVM, we noted that by taking into account training and testing on public dataset, Ac = 88.07% was obtained, whereas considering training on simulated data and testing on public dataset resulted in Ac = 57.3%. Dividing the five classification results into three levels (low, mild, and severe) directly, we found that our proposed model reached Ac = 91.8%, which is higher than that of baseline model (Ac = 85.7%). Specially, the measures of low (Se = 92.9%, Sp = 96.1%), mild (Se = 89.1%, Sp = 93.9%), and severe (Se = 85.6%, Sp = 97.5%) were obtained by our proposed model. In contrast, baseline model merely resulted in Se = 86.3%, Sp = 89.9% (low), Se = 82.2%, Sp = 88.5% (mild), and Se = 87.2%, Sp = 96.9% (severe). Furthermore, Table 6 shows the accuracy results obtained on public dataset per record. It can be seen that good Ac was achievable when the dominant rhythm was sinus rhythm. However, the Ac was lower when arrhythmias were atrial flutter (AFL) and atrial fibrillation (AF) (record 202, 203, 210, 221, and 222) and ventricular flutter (VFL) (record 207). This reveals that the irregular arrhythmia waveforms, such as AFL/AF waves and VFL waves, which are similar to myoelectrical noise to a certain degree, will impact signal quality classification.

### 3.5. Discussions

#### 3.5.1. Comparisons with Previous Studies

The highest ECG acceptability accuracy listed in Table 5 and reported for the PCinC challenge is 94.6% [3]. While this approach is applicable to dynamic ECG signals, clinical use requires more specific interference level classification. Li et al. divided ECG noise into five levels including clean (no visible noise or artifact), minor noise (transient artifacts or low-level noise that does not interfere with the interpretation or recognition of P, T, or atrial flutter waves), moderate noise (interpreted with confidence despite visible and obvious flaws—does not interfere with recognition of QRS complexes or ventricular flutter waves), severe noise (interpretable with difficulty—noise interferes with QRS), and extreme noise (unacceptably poor recording that cannot be interpreted) [10]. They extracted 13 features including bSQI (the percentage of beats detected by *wqrs* that were also detected by *eplimited* [36]), sSQI (the skewness of the ECG signal), kSQI (the kurtosis of the ECG signal), pSQI (the relative power in the QRS complex), basSQI (the relative power in the baseline), bsSQI (the baseline wander check in the domain), eSQI (the relative energy in the QRS complex), hfSQI (the relative amplitude of high frequency noise), purSQI (signal purity of ECG), rsdSQI (the relative standard deviation (STD) of QRS complex wave), entSQI (the sample entropy of the ECG waveform), hfMSQI (high-frequency mask of ECG waveform), and PicaSQI (periodic component analysis (PiCA) periodicity measure of the ECG waveform). Although this annotation is considered fine-grained, based on its signal-to-noise ratio (SNR), it neglects the characteristics of different interference types and uses complex nonlinear features. A support vector machine (SVM) was trained and validated on the MIT-BIH set (88.07%). However, this approach offers poor generalizability because of limited individuals. In contrast, our model labels more data, does not require feature engineering, has powerful nonlinear mapping capabilities, and offers better generalizability. It is worth mentioning that three levels (low, mild, and severe) effectively meet clinical requirements for dynamic ECGs as well.

#### 3.5.2. Effect of the Cascaded Model

With regards to the experiment results, it is important to note that our proposed model was superior to the baseline model and had advantages over the previous studies. While using the baseline model for classifications, we came across an awkward problem that mild noise and severe noise were classified somewhat mistakenly, which led to a lower Se and Sp. For example, Se = 85.8% (mild motion artifacts) and Se = 88.0% (severe motion artifacts) were only obtained by the baseline model provided in Table 4. Besides, we discovered that the three interference types (low interference, myoelectrical noise, and motion artifacts) have distinguishable characteristics, as described in Section 1. The interference levels (mild myoelectrical noise, severe myoelectrical noise, mild motion artifacts, and severe motion artifacts) have somewhat potential characteristics which should be deeply mined in time and frequency domain, as shown in Figure 4. Therefore, instead of classifying ECG signals into five classifications using a baseline model directly, our cascaded CNN model exploited the powerful ability of learning feature representations, firstly evaluating the types of ECG signals and secondly assessing levels of signal quality via two binary tasks.

#### 3.5.3. Efficiency

The number of trainable parameters in the baseline model was 0.28 million, compared with 3.2 million in the proposed model. This is primarily due to the use of time-frequency spectra and network cascades. The use of high-performance computers makes this process more efficient.

Interference categorization is an important part of computer interpretation, more so than the end result. It is evident that our proposed model should only be used for preliminary screening in clinical applications, after which a clinician should perform manual corrections. As such, we provide guidelines for clinicians to validate and correct these prediction results (if need be). The highest interference levels should be assigned to dynamic ECG segments. Specifically, if a prediction result includes low interference and mild interference lasting less than 2 seconds, it should be classified as mild interference. Similarly, if a result includes low interference and severe interference lasting less than 2 seconds, it should be classified as severe interference. If a result includes mild interference and severe interference lasting less than 2 seconds, it should be classified as severe interference. The application of this model has the potential to improve clinical diagnostic efficiency, allowing physicians to focus on diagnosing dynamic ECG segments for different applications, such as arrhythmia, myocardial ischemia, heart rate, HRV, and so on.

## 4. Conclusion

This study addressed the shortage of quality assessment methods for dynamic ECG signals. The proposed cascaded CNN containing two stages divided the signals into three levels including low, mild, and severe, to meet the needs of clinical requirements. Motivated by the fact that three categories are comparatively distinguishable, the first stage was capable of classifying dynamic ECG signals into low interference, myoelectrical noise, and motion artifacts. The second stage estimated the degree of signal interference, primarily using CNN feature representations to conduct binary classification. Results showed the overall recognition accuracy for the model reached 92.7% on private dataset. It is important to note that an overall accuracy of 91.8% was obtained by our proposed model on public dataset. These results suggest the proposed method to be a valuable new tool for dynamic ECG auxiliary diagnosis. Future studies will aim to further increase the model accuracy and compress the model without lowering the accuracy.

## Figures and Tables

**Table 1:** Signal quality of lead II in the MIT-BIH arrhythmia database.

Class\database	MIT-BIH arrhythmia
# Low interference	16419
# Mild myoelectrical noise	2373
# Mild motion artifacts	1512
# Severe myoelectrical noise	432
# Severe motion artifacts	864
Total	21600

**Table 2:** The baseline model configuration.

Baseline model
Input (1 × 512)
↓
W1_block2_32
↓
Max_pooling_1 × 4
↓
W2_block2_64
↓
Max_pooling_1 × 4
↓
W3_block2_128
↓
Max_pooling_1 × 4
↓
Conv_1 × 1_32
↓
Fc_128
↓
Softmax

Vertical arrows indicate sequential connections in the network. Note that W1, W2, and W3 are the number of block2 items. For example, W1_block2_32 denotes that W1 block2 items are connected sequentially, each of which outputs 32 feature maps. Max_pooling_1 × 4 denotes a max pooling with a size of 1 × 4.

**Table 3:** The configuration of CNN1 and CNN2 in the dynamic ECG signal quality assessment model.

CNN1	CNN2
Input (257 × 63)	Input (1 × 512)
↓	↓
N1_block1_32	N1_block2_32
↓	↓
Max_pooling_4 × 2	Max_pooling_1 × 4
↓	↓
N2_block1_64	N2_block2_64
↓	↓
Max_pooling_4 × 2	Max_pooling_1 × 4
↓	↓
N3_block1_128	N3_block2_128
↓	↓
Max_pooling_4 × 4	Max_pooling_1 × 4
↓	↓
Conv_1 × 1_32	Conv_1 × 1_32
Fc_128 (dropout)	
Softmax	

The vertical arrows indicate sequential connections in the network. Note that N1, N2, and N3 are the number of blocks. N1_block1_32 indicates that N1 block1 items were connected sequentially, each of which outputs 32 feature maps. Max_pooling_4 × 2 denotes a max pooling with a size of 4 × 2.

**Table 4:** Classification results of baseline and cascaded CNN model on private dataset.

Class\model	Baseline			Cascaded CNN		
	Se (%)	Sp (%)	Ac (%)	Sp (%)	Sp (%)	Ac (%)
Low interference	90.3	96.2		95.4	98.4	
Mild myoelectrical noise	87.9	97.3		91.1	98.2	
Severe myoelectrical noise	89.5	97.6	88.3	92.9	98.4	92.7
Mild motion artifacts	85.8	96.5		92.3	97.7	
Severe motion artifacts	88.0	97.6		91.8	98.3	

**Table 5:** Comparisons with previous studies on public dataset.

Model	7-SQI SVM [3]	13-SQI SVM [10]^*∗*^					13-SQI SVM [10]^+^	Baseline			Cascaded CNN		
Level	Bad or good	Clean	Minor	Moderate	Severe	Extreme	—	Low	Mild	Severe	Low	Mild	Severe
Se (%)	86.3	56.9	59.3	56.2	46.7	45.3	—	86.3	82.2	87.2	92.9	89.1	85.6
Sp (%)	94.8	92.5	65.9	87.9	97.1	99.1	—	89.9	88.5	96.9	96.1	93.9	97.5
Ac (%)	94.6		57.3				88.07		85.7			91.8	

^*∗*^Trained on simulated dataset and tested on public dataset; SVM with 13 signal quality assessment indices (SQIs) classified ECG signals into five levels (clean, minor, moderate, severe, and extreme noise). ^+^SVM with 13 SQIs was trained and tested on public dataset, for which only accuracy of five classifications was given.

**Table 6:** Results per record on public dataset using cascaded CNN model.

Record	Dominant rhythm	Ac (%)	Record	Dominant rhythm	Ac (%)	Record	Dominant rhythm	Ac (%)
100	N	94.7	117	N	97.6	212	N	95.3
101	N	96.4	118	N	96.0	213	N, B	96.7
102	P	93.6	119	N, B, T	93.1	214	N, T	91.8
103	N	96.2	121	N	96.4	215	N	92.4
104	P	95.3	122	N	97.1	217	P, AF	82.7
105	N	95.8	123	N	96.4	219	N, AF	79.3
106	N, B	93.6	124	N	95.8	220	N	96.7
107	P	94.2	200	N, B	95.3	221	AF, T	78.9
108	N	96.7	201	N, AF, T	80.2	222	N, AFL, NOD, AB	77.3
109	N	94.7	202	N, AF	79.6	223	N, B, VT, T	94.0
111	N	93.6	203	AFL, AF	75.1	228	N, B	93.6
112	N	93.1	205	N	94.9	230	N, PREX	94.4
113	N	92.9	207	N, VFL, SVTA, B, IVR	77.6	231	N, BII	95.3
114	N	94.9	208	N, T	96.4	232	SBR	94.0
115	N	94.2	209	N, SVTA	90.4	233	N, B	93.1
116	N	96.2	210	AF	80.9	234	N, SVTA	92.0

N, normal sinus rhythm; AB, atrial bigeminy; AFL, atrial flutter; AF, atrial fibrillation; B, bigeminy; BII, 2nd-degree heart block; IVR, idioventricular rhythm; NOD, nodal rhythm; P, paced rhythm; PREX, preexcitation (WPW); SBR, sinus bradycardia; SVTA, supraventricular tachyarrhythmia; T, ventricular trigeminy; VFL, ventricular flutter; VT, ventricular tachycardia.

## Data Availability

The data used to support the findings of this study are available from the corresponding author upon request.
